# Engineered exosomes enriched in netrin-1 modRNA promote axonal growth in spinal cord injury by attenuating inflammation and pyroptosis

**DOI:** 10.1186/s40824-023-00339-0

**Published:** 2023-01-17

**Authors:** Xiao Lu, Guangyu Xu, Zhidi Lin, Fei Zou, Siyang Liu, Yuxuan Zhang, Wei Fu, Jianyuan Jiang, Xiaosheng Ma, Jian Song

**Affiliations:** 1 Department of Orthopedics, Huashan Hospital, Fudan University, No. 12, Middle Wulumuqi Road, Jing’an District, Shanghai, 200040 China; 2 Institute of Pediatric Translational Medicine, Shanghai Children’s Medical Center, School of Medicine, Shanghai Jiao Tong University, Shanghai, 200127 China

**Keywords:** Engineered exosomes, Netrin-1, modRNA, Spinal cord injury, Unc5b/PI3K/AKT/mTOR

## Abstract

**Background:**

Spinal cord injury (SCI) brings a heavy burden to individuals and society, and there is no effective treatment at present. Exosomes (EX) are cell secreted vesicles containing molecules such as nucleic acids and proteins, which hold promise for the treatment of SCI. Netrin-1 is an axon guidance factor that regulates neuronal growth. We investigated the effects of engineered EX enriched in netrin-1 chemically synthetic modified message RNA (modRNA) in treating SCI in an attempt to find a novel therapeutic approach for SCI.

**Methods:**

Netrin-1 modRNA was transfected into bone marrow mesenchymal stem cells to obtain EX enriched with netrin-1 (EX-netrin1). We built an inflammatory model in vitro with lipopolysaccharide (LPS) in vitro to study the therapeutic effect of EX-netrin1 on SCI. For experiments in vitro, ELISA, CCK-8 assay, immunofluorescence staining, lactate dehydrogenase release experiments test, real-time quantitative polymerase chain reaction, and western blot were conducted. At the same time, we constructed a rat model of SCI. MRI, hematoxylin-eosin and Nissl staining were used to assess the extent of SCI in rats.

**Results:**

In vitro experiments showed that EX had no effect on the viability of oligodendrocytes and PC12 cells. EX-netrin1 could attenuate LPS-induced inflammation and pyroptosis and accelerate axonal/dentritic growth in PC12 cells/oligodendrocytes. In addition, netrin-1 could activate the PI3K/AKT/mTOR signalling pathway upon binding to its receptor unc5b. When Unc5b and PI3K were inhibited, the effect of EX-netrin1 was weakened, which could be reversed by PI3K or mTOR activator. Our in vivo experiments indicated that EX-netrin1 could promote recovery in rats with SCI.

**Conclusion:**

We found that EX-netrin1 regulated inflammation, pyroptosis and axon growth in SCI via the Unc5b/PI3K/AKT/mTOR pathway, which provides a new strategy for the treatment of SCI.

**Supplementary Information:**

The online version contains supplementary material available at 10.1186/s40824-023-00339-0.

## Introduction

As a common disease in spinal surgery, spinal cord injury (SCI) refers to a central nervous system catastrophe mainly characterized by abnormal physiological function or absence of sensation, movement and other functions caused by complete or incomplete injury after direct or indirect violence on the spinal cord tissue [1, 2]. According to statistics, there are approximately 10.4–83 cases of SCI in one million people every year worldwide, and its incidence and mortality are also increasing year by year, which creates many difficult problems in medical fields [3]. Unfortunately, regeneration after SCI is extremely weak due to disadvantages, such as poor central nervous system plasticity and limited neuronal regeneration capacity. At present, there is no effective treatment to completely restore neural function after spinal cord injury [4].

Exosomes (EXs) are cell-derived nanovesicles involved in intercellular material transport and information transmission with good stability, biocompatibility, and permeability as well as low immunogenicity [5]. Genetically engineered cells can impart novel functions to EXs by providing EXs with functional proteins [6, 7]. In addition, EXs can be applied as vehicles containing small molecules or nucleic acid drugs for targeted drug delivery into specific types of cells or tissues [8–10]. Many studies have confirmed that engineered EXs hold promise for the treatment of SCI [11–13].

Netrin-1 is one of the first recognized axon guiding factors and plays a certain role in axon guiding growth [4]. By specific binding to its receptors, it can initiate axon growth and branch formation, [14, 15] facilitate the migration and survival of neurocytes, [16, 17] and inhibit neuronal apoptosis [18]. A recent study demonstrated that netrin-1 acts with Unc5b to attenuate cerebral ischaemic injury and promote angiogenesis and neurological functional recovery [19]. In addition, netrin-1 can activate the phosphatidylinositol-3-kinase (PI3K)/protein kinase B (PKB or AKT)/mammalian target of the rapamycin (mTOR) pathway through unc5b [20]. This axis plays a vital role in promoting axonal growth and mitigating inflammation [21, 22].

Chemically synthetic modified message RNA (modRNA), as a novel gene vector, refers to mRNA synthesized in vitro using chemically modified bases. This technology takes different modified bases as raw materials and transcribes DNA fragments containing mRNA biological information in vitro to synthesize modRNA. Then, modRNAs are transferred into target cells through liposomes or nanoparticles for protein translation and expression to achieve rapid, efficient and pulse expression of bioactive target proteins in vivo and in vitro. They thereby exert therapeutic effects [23]. Compared with the conventional plasmid or viral gene vector, modRNA has the characteristics of high safety, [24] strong controllability, good stability, low immunogenicity and high protein translation ability [25–27].

Based on the above evidence, engineered EXs originating from bone marrow mesenchymal stem cells (MSCs) containing netrin-1 modRNA were constructed in the current study to investigate their therapeutic value in SCI through in vivo and in vitro experiments. In addition, the PI3K/AKT/mTOR axis has been reported to take part in the regulation of pyroptosis in a variety of diseases, [28] but its association with Unc5b and SCI has not been studied. Thus, this study investigated whether netrin-1 influences the progression of SCI by regulating the PI3K/AKT/mTOR axis through Unc5b.

## Methods and materials

### Isolation and culture of bone marrow mesenchymal stem cells (MSCs)

The animal experiments in this study were approved by the Research Ethics Committee of School of Medicine, Fudan University (No. 202203011S). MSCs were extracted from the femur and tibia of six Sprague–Dawley (SD) rats, and the bone marrow cavity was washed with DMEM-LG (GIBCO, US) and then filtered with a 200-mesh nylon filter. DMEM containing 10% foetal bovine serum and 1% penicillin was used to culture MSCs in a constant temperature incubator at 37 °C and 5% CO2 (SCO6WE, SHEL LAB). The second- or third-generation cells were collected and used for subsequent analysis and experiments. MSC surface marker assays were performed by flow cytometry (FACSCalibur, BD Biosciences), and the cells were positive for CD73, CD90 and CD105 but negative for CD34 and CD45.

### Chemically synthetic modified message RNA (modRNA) synthesis and transfection

**Fig. 1:**
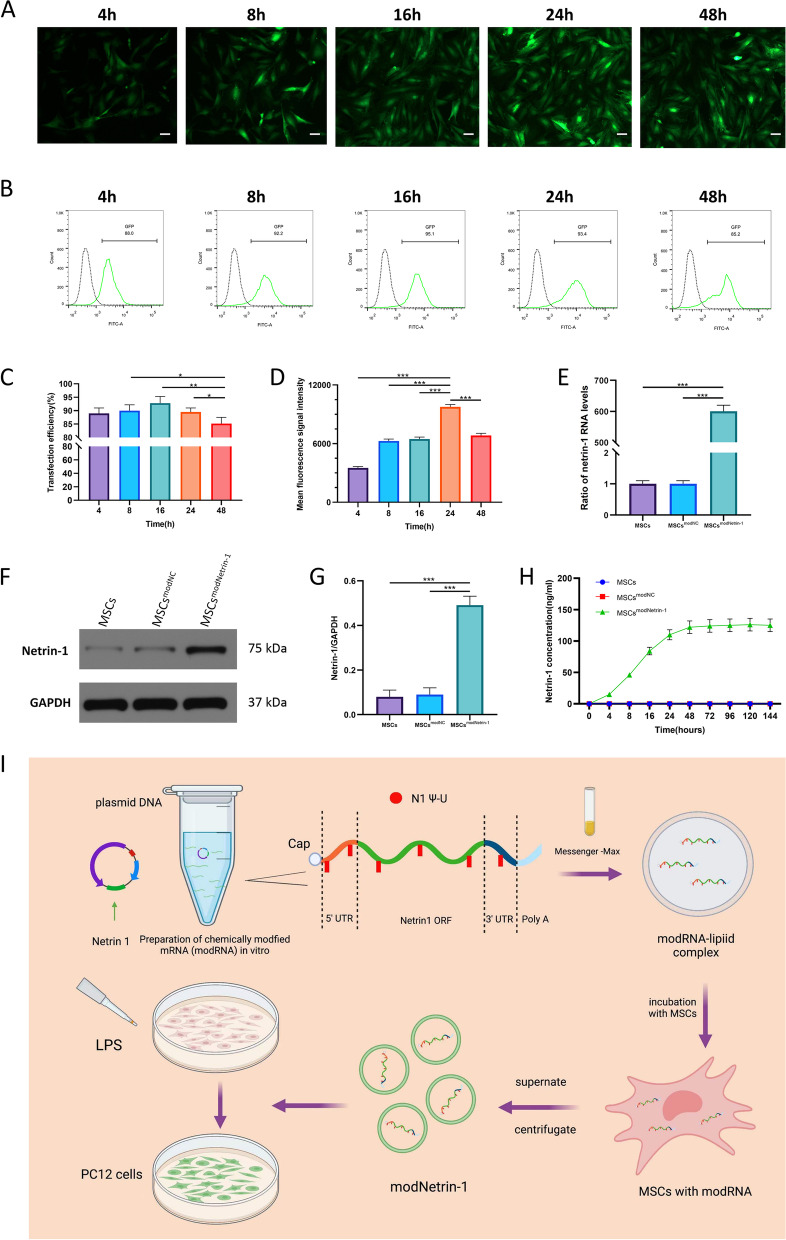
Efficiency and kinetics of modRNA transfection in MSCs. (**A**–**D**) Transfection efficiency and the expression kinetics of modGFP in MSCs. (**A**) Representative images depicting GFP signal in MSCs at 4, 8, 16, 24, and 48 h post-transfection. Scale bar = 100 μm. (**B**-**C**) Flow cytometry analysis of transfection efficiency at 4, 8, 16, 24, and 48 h post-transfection. (D) Flow cytometry analysis of mean fluorescence signal intensity at 4, 8, 16, 24, and 48 h post-transfection. (**E**-**G**) Expression levels of (**E**) netrin-1 mRNA and (**F**–**G**) netrin-1 protein at 24 h post-transfection. (H) Kinetics of cumulative netrin-1 protein concentrations periodically monitored for several days following transfection of modNetrin-1 in MSCs. (**I**) Research flow for in vitro experiments. First, the modRNA was synthesized and transfected into MSCs. Then the engineered exosomes rich in Netrin-1 modRNA were isolated. Finally, observed the therapeutic effect of exosomes on LPS induced PC12 cells. (Error bars showed means ± SD; n = 3; *p < 0.05, **p < 0.01, ***p < 0.001)

We synthesized and transfected modRNA as previously described [29]. In brief, mRNA was first synthesized in vitro using chemically modified bases and then transferred into target cells via mediators, such as liposomes or nanoparticles (Fig. 1I).

### Quantitative real-time polymerase chain reaction (qRT–PCR)

TRIpure reagent (ELK Biotechnology, EP013) was used to extract total RNA from spinal cord tissues, MSCs, exosomes and PC12 cells. The following primers were used: netrin-1, forward primer, 5′-GTCTGAGAACTACCTGCAGTTCC-3′ and reverse primer, 5′-TACATTTTGCGGCACTGAGTG-3′; GAPDH, forward primer, 5′-AACAGCAACTCCCATTCTTCC-3′ and reverse primer, 5′- TGGTCCAGGGTTTCTTACTCC-3′. GAPDH was used for normalization.

### Western blot (WB) analysis

Total protein was extracted from cells or tissues using a whole protein extraction kit (AS1012, ASPEN). The primary antibodies (ASPEN) used were netrin-1 (1:1000), CD63 (1:2000), CD9 (1:2000), TSG101 (1:1000), nucleotide-binding oligomerization domain-like receptor protein 3 (NLRP3) (1:1000), apoptosis-associated speck-like protein (ASC) (1:1000), pro-caspase-1 (1:2000), cleaved caspase-1 (1:2000), pro-IL-1β (1:2000), IL-1β (1:2000) (Abcam), Unc5b (1:2000), PI3K (1:2000), p-PI3K (1:2000), Akt (1:2000), p-Akt (1:2000), mTOR (1:2000), p-mTOR (1:2000) and GAPDH (1:2000) (26,616, Thermo). A gel image processing system (gel Pro analyser software) was used to analyse the grey value of the target strip.

### Exosome (EX) isolation from transfected MSCs

MSCs were inoculated into DMEM containing 10% foetal bovine serum (141,215, Tianhang) without exosomes and cultured in a constant temperature incubator at 37 °C and 5% CO2 for 24 h. We collected 40 ml cell culture supernatant, 4 °C 3000×g centrifuged it for 15 min, transferred the supernatant into a new centrifuge tube, mixed the centrifuged cell supernatant with Total Exosome Isolation reagent (Invitrogen ) [30], turned over and mixed evenly for 50 times, and left it in a 4 °C refrigerator overnight for approximately 14 h; then, at 4 °C, it was 3500×g centrifuged for 50 min, and we discarded the supernatant after centrifugation, resuspended with 1 mL of PBS (AS1044, ASPEN), 4 °C 10000×g centrifuged it for 10 min, discarded the supernatant, and then, used 200 μL of PBS to resuspend it; the supernatant was the exosome diluent. Exosome concentration was estimated by total protein level quantified using a BCA Protein Assay Kit (Key-GEN). The concentration is about 20 μg/mL.

### Uptake of EX by PC12 cells

To observe the absorption of exosomes by PC12 cells, we labelled exosomes with PKH26 (MX4021, MKBio) and incubated them with PC12 cells for 1 or 3 hours. The exosomes were labelled with red fluorescence, and the nuclei were stained blue with 4–6-diamidino-2-phenylindole (DAPI) (D8417-1MG, Sigma). A fluorescence microscope (IX51, Olympus; MicroPublisher, Q-IMAGING) was used for image capture at different time points.

### Cell viability assay

PC12 cells were seeded into 96 well plates, and at the end of treatment, 20 μL CCK8 solution (#bs350b, Biosharp) was added to each well and incubated for 4 h, and the absorbance at 450 nm was measured by a microplate reader.

### RNA interference

Inhibition of Unc5b expression in PC12 cells was performed using small interfering RNA (siRNA). Lipofectamine 3000 (Invitrogen) was used to transfect 100 nmol/L Unc5b siRNA into PC12 cells. The siRNA sequences were 5′-TGGACCGGTACCTGAATTA-3′ and 5′-GGACGCCTACATTGTGAAG-3′.

### Cell culture and grouping

PC12 cells are rat adrenal pheochromocytoma cells. After inducing cell differentiation by nerve growth factor (NGF), PC12 cells have neural cell properties and are a commonly used cell line to study neural development and function [31]. Because PC12 cells have neuron like physiological characteristics, we mainly chose PC12 cells instead of spinal neurons for in vitro cell experiments in this study. PC12 cells used in this study were all treated with 5 μg/L NGF for 72 h [32]. Lipopolysaccharide (LPS), which has been mainly used in experiments to study infection, inflammation or tissue damage, can induce inflammatory factor production, amplify inflammatory responses in the central nervous system and promote the development of neurological injury and neurodegenerative diseases. Therefore, the inflammatory model in vitro can be constructed with LPS [13, 33]. Our study included ten groups: ① Control group: PC12 cells were routinely cultured. ② LPS group: PC12 cells were treated with LPS (5 μg/ml, Sigma–Aldrich, MA, USA) for 12 h [13]. In the ③ LPS + EX-MSCs group, ④ LPS + EX-NC group, and ⑤ LPS + EX-Netrin1 group, PC12 cells were treated with 5 μg/ml LPS for 12 h, and then 10 μg/mL EXs isolated from MSCs, MSCs^modNC^, and MSCs^modNetrin-1^ were used to incubate PC12 cells for 48 h. ⑥ LPS + EX-Netrin1 + si-Unc5b group: PC12 cells incubated with si-Unc5b for 48 h, 5 μg/ml LPS for 12 h and 10 μg/mL EX-Netrin1 for 48 h. ⑦ LPS + EX-Netrin1 + si-Unc5b + 740 Y-P group: PC12 cells incubated with si-Unc5b for 48 h, 5 μg/ml LPS for 12 h, 10 μg/mL EX-Netrin1 for 48 h, and 740 Y-P (50 μg/mL, TocrisBioscience, Ellisville, MO, USA) for 24 h [34]. ⑧ LPS + EX-Netrin1 + LY294002 group: PC12 cells cultured with 5 μg/ml LPS for 12 h, 10 μg/mL EX-Netrin1 for 48 h, and LY294002 (10 μM, Beyotime, Haimen, China) for 24 h, a PI3K inhibitor. ⑨ LPS + EX-Netrin1+ LY294002+ MHY1485 group: PC12 cells were cultured with 5 μg/ml LPS for 12 h, 10 μg/mL EX-Netrin1 for 48 h, 10 μM LY294002 for 24 h, and MHY1485 (100 nmol/L, Beyotime, Haimen, China) for 24 h, an activator of mTOR. ⑩ EX-Netrin1 group: PC12 cells were cultured with 10 μg/mL EX-Netrin1 for 48 h.

We also studied the effect of EX on microglia. Microglial cells were obtained from neonatal (about 3-day-old) SD rats, as described previously [35]. Microglial cells were cultured in DMEM supplemented with 3% FBS, penicillin-streptomycin (100 U/ml–100 μg/ml) and 4 mM L-glutamine. Cells were grown on poly-L-lysine coated slides in 6-well plates.

### Enzyme-linked immunosorbent assay (ELISA)

To measure the secreted levels of netrin-1, TNF-α, IL-1β and IL-6 under different conditions, the supernatant was collected for detection. Four ELISA kits were obtained from ELK Biotechnology (ELK5693, ELK1272, ELK1396 and ELK1158). The manufacturer’s instructions were followed for this procedure.

### Immunofluorescence analysis

The cells were fixed with 4% paraformaldehyde (80,096,618, Sinopharm) for 10 min, incubated in PBS (GNM20012, Genom) containing 0. 25% Triton X-100 (V900483, Sigma) for 10 min, incubated with goat serum (4240GR250, Biofroxx) for 30 min, incubated overnight with primary antibodies against Actin (1:300) (ab200658, Abcam) at 4 °C, incubated with fluorescent secondary antibody for 1 h, rinsed again, stained and sealed with DAPI, and the cell morphology was observed by fluorescence microscopy (Eclipse Ci-L, Nikon). In each cell, the longest neurite was selected. We randomly selected five cells in the field of view for length measurement [36].

Paraffin sections of rat spinal cord tissue were dewaxed in water and incubated overnight with neurofilament (NF) (Cell Signaling Technology, USA) and glial fibrillary acidic protein (GFAP) (Boster, China). The secondary antibody was incubated at room temperature at a 1:200 dilution ratio for 90 min. DAPI was added to counterstain the nucleus. After sealing with fluorescent quenching agent, the colocalization of NF and GFAP in spinal cord tissue was observed under a 400× times microscope, and the number of caspase 3-positive cells was analysed [29].

### Detection of pyroptosis

After pyroptosis occurs, the cell membrane produces pores, lactate dehydrogenase (LDH) is released from the cell, [37] and the interstitial fluid enters the cell to form vesicles that resemble bubbles in morphology under microscopy [38].

Lactate dehydrogenase (LDH) released into cell culture supernatants after different treatments was assayed using a lactate dehydrogenase cytotoxicity assay kit (Beyotime Biotech, C0016) [39–41]. Cells were seeded into 96-well plates. When the density reached 80–90%, cells were treated with different drugs at different schedules. Then 120 μl of culture medium was transferred into a new 96-well plate and mixed with 60 μl of LDH work buffer. After 30 min in the dark at room temperature, the absorbance (Optical density, OD) was measured at the wavelength of 490 nm. The percentage of LDH release was calculated according to the following formula: percentage of LDH release = (OD_experimental group_ − OD_control group_)/(OD_max_ − OD_control group_) × 100%. In addition, pyroptosis-associated proteins were tested by WB.

### Construction of a rat model of spinal cord injury

After the rats were adaptively fed for one week, they were anaesthetized by intraperitoneal injection of a mixture of 5 mg/kg xylazine and 70 mg/kg ketamine [42]. Then, with T10 lamina as the centre, the skin was cut, the paravertebral muscles were passively stripped, T9–11 spinous processes and lamina were exposed, T10 spinous processes and lamina were bitten, and the exposed spinal cord was impacted from a 3-cm height with a 20-g impactor to produce moderate contusion injury. Contusion at the injured site, convulsions of both lower limbs and spasmodic swing of the tail were signs of successful modelling. Bladder massage was performed twice a day to prevent urinary system infection until spontaneous urination [11].

### Grouping of animal experiments

Forty-eight 4-week-old SD female rats were randomly assigned to six groups: Sham, Model, Model+PBS, Model+EX-MSCs, Model+EX-NC, and Model+EX-Netrin1 groups. Each group contained eight rats. In the sham group, only T10 laminectomy was performed without impact. Before suturing, the last three groups of rats received a tail vein injection with EX [12, 13]. Tail injections were performed for 5 consecutive days, once a day. EX was extracted from MSCs in the MSCs, NC and netrin-1 groups (100 μg of total EX protein was precipitated in 0.5 mL of PBS, equivalent to 1 × 10^10^ particles) [13]. Rats in the Model+PBS group received a tail vein injection with 0.5 mL of PBS. After 28 days, MRI was used to detect the level of SCI.

### Basso, Beattie & Bresnahan (BBB) locomotor rating scale

BBB scoring was performed on each group at each time point of observation (1, 3, 7, 14, 21, and 28 d) to evaluate the status of locomotor recovery [43, 44]. The BBB scores were individually scored three times by two persons and averaged. Scores range from 0 to 21, with lower scores indicating more severe SCI.

### Nissl staining

Rats were sacrificed after 28 days, and spinal cord tissues were collected for subsequent studies. Pathological sections (the steps are the same as HE staining) were made using tar purple (C861450, Macklin, Shanghai, China) as the core dye, washed with ionic water after dyeing for 1 h, and decolorized with differentiation agent. Nissl bodies are dark blue purple particles or patches, with light blue nuclei and a colourless or light blue background.

### Haematoxylin and eosin (HE) staining

Taking the injury point as the centre, the spinal cord tissues within 5 mm longitudinally from the injury point were collected and fixed with 4% paraformaldehyde for 24 h, embedded in paraffin (69,019,461, Sinopharm), and sectioned at 3-μm thickness for HE staining (D12621, Xiya; H9627-25G, Sigma). After washing, drying and resin sealing, the histopathological changes in the spinal cord were observed under a microscope.

### TUNEL and cleaved-caspase1 immunofluorescence double staining

TUNEL (green) and cleaved-caspase1 immunofluorescence (red) double staining were used to evaluate neuronal pyroptosis after SCI. After inhibiting endogenous peroxidase and antigen repair, paraffin sections were stained with TUNEL (11,684,817,910, Roche). Then, cleaved-caspase1 antibody was added to the sections and incubated overnight at 4 °C. Then, the sections were incubated with fluorescent dye-labelled secondary antibodies at room temperature in a dark environment for 2 h. Finally, DAPI stained the nuclei, and anti-fluorescence quenching blocking reagent blocked the sections. The sections were observed and photographed with a fluorescence microscope.

### Statistical analysis

For all in-vitro experiments, *n* = 3 are biological replicates, and the experiments were carried out in at least two independent experiments. SPSS 22.0 (IBM, NY, USA) statistical software was used for analysis. The measurement data are expressed as the means ± standard deviation. When the normal distribution was satisfied (Shapiro-Wilk W test) and the variance was homogeneous, the data between the two groups were compared by t-test, the data between the multiple groups were compared by single factor analysis of variance (ANOVA). When the data does not follow the normal distribution or the variance is uneven, the data between the two groups were compared by Wilcoxon rank-sum test, the data between the multiple groups were compared by Kruskal-Wallis test. The LSD test was used for pairwise comparison. The bilateral inspection level was α = 0.05. The figures were drawn by Figdraw and GraphPad Prism 9.

## Results

### MSCs showed good tolerance to modRNA

We first transfected MSCs with modRNA encoding green fluorescent protein (modGFP) to investigate the kinetics and efficiency of modRNA in MSCs. We found a high tolerance of MSCs to modRNA, with up to 92.8% ± 2.5% transfection efficiency of modGFP at 16 h (Figs. 1B, C). At 24 h after transfection, GFP had the highest mean fluorescence intensity of 9755.0 ± 250 (Figs. 1A, D).

Next, we transfected modRNA encoding netrin-1 (MSCs^modNetrin-1^) and the negative control (MSCs^modNC^) into MSCs to determine the expression of the target gene in MSCs. The level of netrin-1 mRNA in MSCs after transfection with modNetrin-1 was approximately 6 × 10^4^ times higher than that in the control groups, as determined by qRT–PCR (Fig. 1E). In addition, WB assays showed that the expression of intracellular netrin-1 protein in the MSCs^modNetrin-1^ group was approximately 5 times higher than that in MSCs group and MSCs^modNC^ group (Figs. 1F, G). These results indicated that modNetrin-1 could stably exist and be expressed in MSCs.

Since netrin-1 is a secreted protrin, we measured the level of accumulated netrin-1 protein in the supernatant of the culture medium by ELISA. We found that the MSCs^modNetrin-1^ group had significantly increased netrin-1 protein concentrations in the supernatant from 4 to 144 h compared with both control groups (Figs. 1H). Fig. 1I illustrates the research flow for in vitro experiments.

### Isolation and identification of MSC-derived EXs

**Fig. 2:**
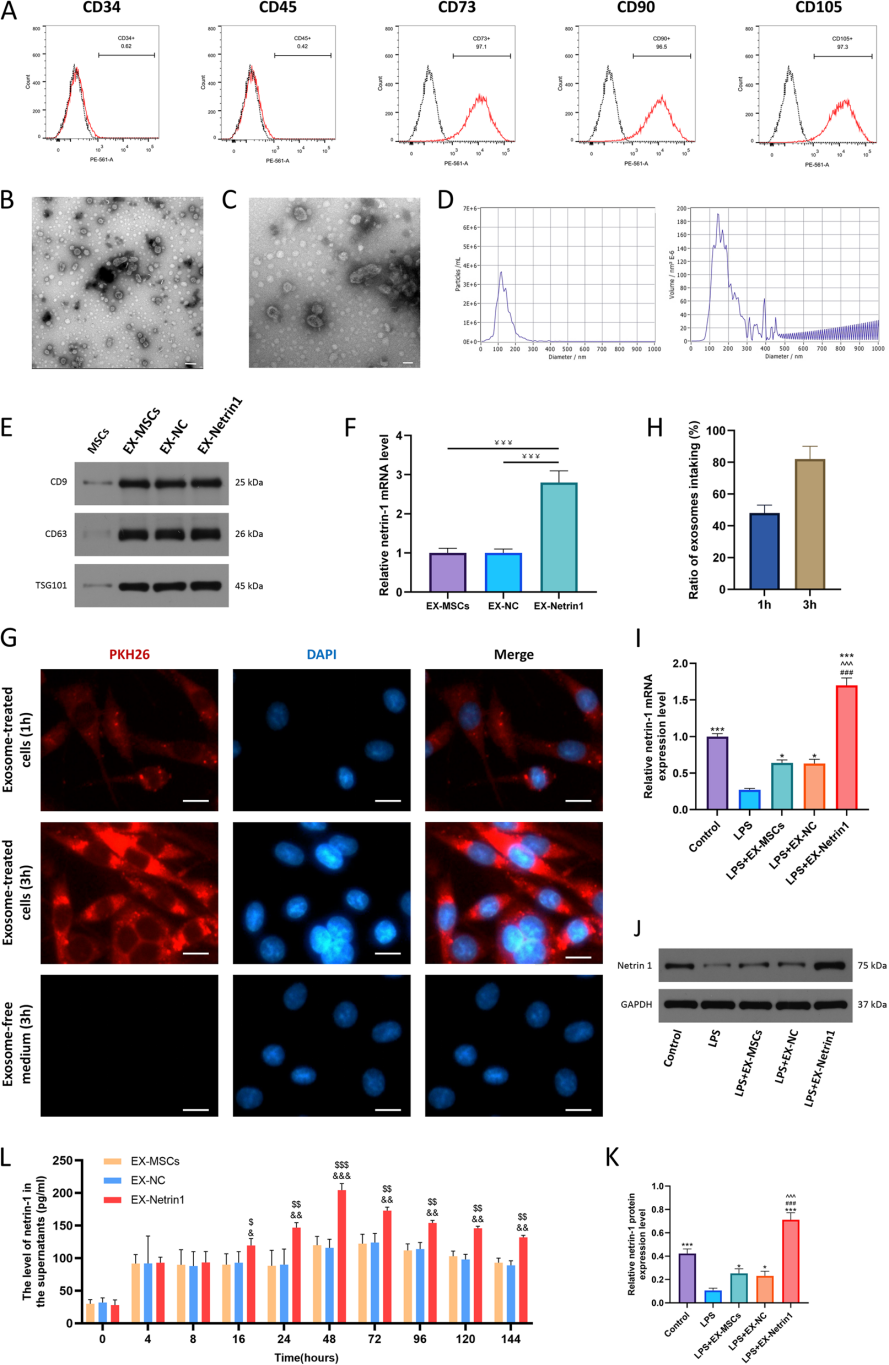
Identification of MSCs and exosomes (EXs), PC12 cells engulfed EXs and expressed netrin-1 protein. (**A**) Cell surface markers (CD34, CD45, CD73, CD70 and CD105) of MSCs was detected by flow cytometric analysis. (**B**-**C**) Typical images of EXs morphology were captured by transmission electron microscope (TEM). Scale bar: (**B**) 200 nm (**C**)100 nm. (**D**) Particle size distribution of EXs was measured by nanoparticle trafficking analysis (NTA). (**E**) Protein markers of EXs were detected by western blot analysis in EXs and MSCs. EX-MSCs: Exosomes derived from MSCs without mRNA transfection; EX-NC: Exosomes derived from MSCs transfected with negative control mRNA; EX-netrin1: Exosomes derived from MSCs transfected with netrin-1 mRNA. (**F**) The relative level of netrin-1 mRNA in three different EX was detected by qRT-PCR. (**G**) A fluorescence microscope was used to observe the changes in the uptake of fluorescently labelled EXs by PC12 cells. Scale bar = 20 μm. (**H**) Percentage of EXs absorbed by PC12 cells at different time points. (I) A cell injury model was constructed by treating PC12 cells with lipopolysaccharide (LPS). qRT-PCR was used to detect the expression of netrin-1 mRNA in PC12 cells. (**J**-**K**) Western blot was used to detect the expression of netrin-1 protein in PC12 cells. (**L**) The levels of netrin-1 in the supernatants of PC12 cells treated with three different EXs at different time points were identified by ELISA. (Error bars showed means ± SD; n = 3; ^￥￥￥^p < 0.001; *P < 0.05, ***P < 0.001, vs. LPS group; ^###^P < 0.001, vs. LPS+EX-MSCs group; ^^^P < 0.001, vs. LPS+EX-NC group; ^$^P < 0.05, ^$$^P < 0.01, ^$$$^P < 0.001, vs. EX-MSCs group; ^&^P < 0.05, ^&&^P < 0.01, ^&&&^P < 0.001, vs. EX-NC group)

There were specific antigens on the surface of MSCs, and flow cytometric detection found that MSCs possessed CD73, CD90 and CD105 but not CD34 and CD45 (Fig. 2A), which was consistent with previous findings [30]. Then, we determined that vesicles isolated from the medium of MSCs were EXs by morphology, size and surface markers. Transmission electron microscopy (TEM) showed that these vesicles had the morphology of EX, as goblet or spherical (Figs. 2B, C). Nanoparticle trafficking analysis (NTA) showed that the vesicle diameter was mainly distributed between 30 and 200 nm (Fig. 2D). WB showed that these vesicles had specific surface markers, such as TSG101, CD9, and CD63 (Fig. 2E), which was consistent with previous study [13, 29, 44, 45]. Finally, we detected the content of netrin-1 mRNA in EXs from the untransfected MSCs (EX-MSCs), MSCs^modNC^ (EX-NC) and MSCs^modNetrin-1^ (EX-Netrin1) groups. qRT–PCR revealed significantly higher netrin-1 mRNA level in the EX-Netrin1 group than in the EX-MSCs group and EX-NC group (Fig. 2F).

### PC12 cells engulfed EXs and expressed netrin-1 protein

We used PKH26 to label EXs to explore whether PC12 cells could take up EXs. The results showed that approximately 80% of PC12 cells had fluorescence in the culture medium after EX treatment compared to the medium without EX after 3 h of incubation (Figs. 2G, H). This shows that PC12 cells have a high EXs absorption efficiency. We investigated the therapeutic value of EX-netrin1 on SCI by in vitro experiments. Inflammatory model in vitro was constructed by lipopolysaccharide (LPS), which was used for the following experiments. We found that LPS could significantly reduce the viability of PC12 cells and microglial cells, while the EXs had no obvious toxicity to the cell viability, and could save the decreased cell viability caused by LPS (Fig. S1). In addition, we found that EX-Netrin1 promoted the activation of PC12 cells. The expression level of microtubule-associated protein 2 (MAP2) in PC12 cells was significantly increased after EX-Netrin1 treatment (Fig. S2).

After different treatments, we detected the expression of netrin-1 in cells of each group by qRT–PCR (Fig. 2) I and western blot (Figs. 2 J, K). According to the results, LPS reduced the level of netrin-1, and this effect could be weakened by EXs, especially by EX-netrin1. Next, the level of netrin-1 in the supernatant of the culture medium was measured by ELISA, and the amount of secreted netrin-1 protein was markedly increased by EX-netrin1 over time (Fig. 2 L). The above results demonstrated that the engineered EX-netrin1 could effectively and stably improve netrin-1 expression.

### EX-Netrin1 alleviated LPS-induced inflammation and pyroptosis in PC12 cells, promoted nerve recovery, and upregulated the PI3K/Akt/mTOR pathway

**Fig. 3:**
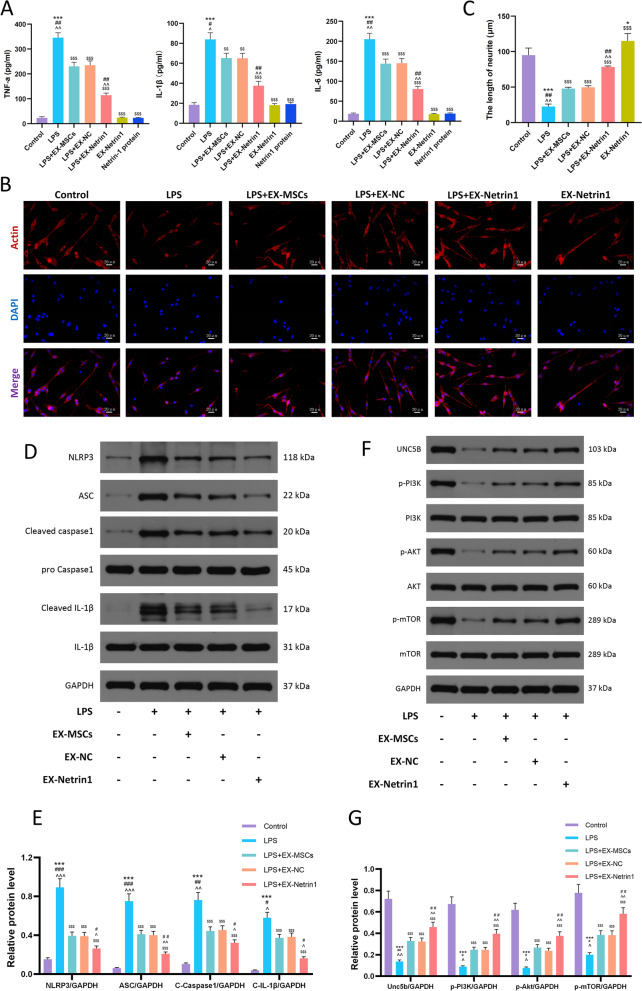
EX-Netrin1 alleviated LPS-induced inflammation and pyroptosis of PC12 cells, promoted nerve recovery, and upregulated the PI3K/Akt/mTOR pathway. (**A**) ELISA was performed to detect the contents of TNF-α , IL-1β and IL-6 in the cell supernatant. (**B**-**C**) Fluorescence microscope was used to take representative images of neuronal cells and measure axonal length. Actin was stained red and nuclei were stained blue with DAPI. Scale bar: 20 μm. (**D**-**E**) Detection of pyroptosis-related proteins by western blot. (**F**-**G**) The PI3K/Akt/mTOR pathway-related proteins were detected by western blot. (Error bars showed means ± SD; n = 3; *P < 0.05, **P < 0.01, ***P < 0.001, vs. Control group; ^#^P < 0.05, ^##^P < 0.01, ^###^P < 0.001, vs. LPS+EX-MSCs group; ^P < 0.05, ^^P < 0.01, ^^^P < 0.001, vs. LPS+EX-NC group; ^$^P < 0.05, ^$$^P < 0.01, ^$$$^P < 0.001, vs. LPS group)

We first detected inflammatory factors in the cell supernatant by ELISA. Figs. 3 illustrates that EXs can lessen the levels of TNF-α, IL-1β and IL-6 in the inflammatory model in vitro, in which the effect of EX-Netrin1 was more obvious. And there was no significant difference in the levels of inflammatory factors between the EX-Netrin1 group, netrin-1 protein (10 μg/mL) treated group and the control group. We next measured the axonal length of cells in different groups using fluorescence microscopy, which is an important indicator for assessing the degree of neural recovery. Figs. 3B-C demonstrate that under LPS treatment of, the axons were shortened to 22.72 ± 3.28 μm, while EXs alleviated this injury, and EX-Netrin1 restored axon length to 78.27 ± 1.58 μm. Pyroptosis-associated proteins, including NLRP3, ASC, pro-caspase-1, cleaved-caspase-1, pro-IL-1β, and cleaved-IL-1β, were detected by WB. The level of pyroptosis was notably increased by LPS, which was mitigated by EXs, and the best effect was achieved by EX-Netrin1 (Figs. 3D, E). EX-Netrin1 had a similar effect on microglia in vitro (Fig. S3). We further tested the PI3K/Akt/mTOR signalling pathway by WB. On the one hand, the number of Unc5b receptors was markedly decreased by LPS and increased by EX-netrin1; on the other hand, LPS inhibited the phosphorylation level of the PI3K/Akt/mTOR axis, which was elevated by EX-netrin1 (Figs. 3F, G).

### The therapeutic effect of EX-Netrin1 was diminished by a PI3K inhibitor but reversed by a mTOR agonist

**Fig. 4:**
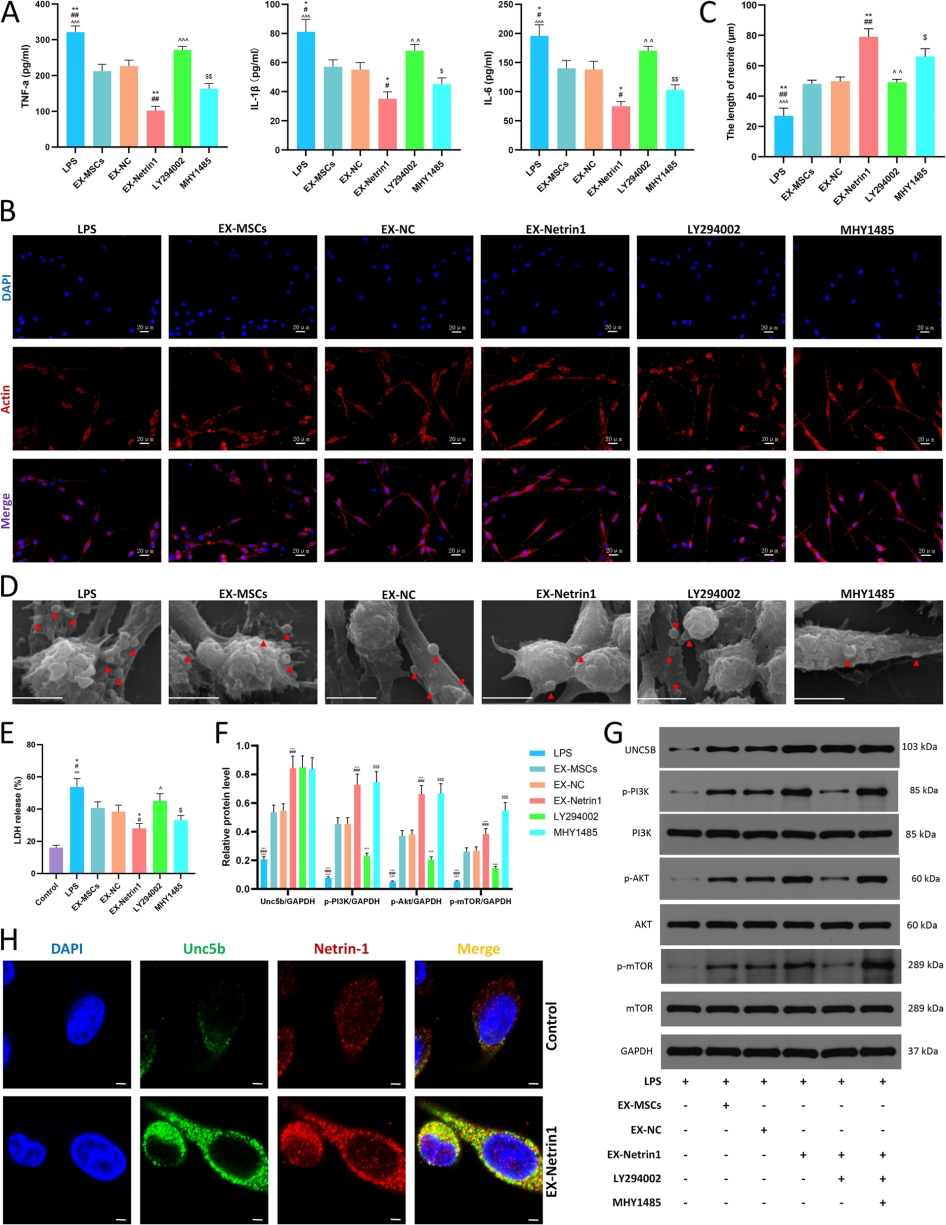
The therapeutic effect of EX-Netrin1 was diminished by a PI3K inhibitor but reversed by a mTOR agonist. (**A**) ELISA was performed to detect the contents of TNF-α , IL-1β and IL-6 in the cell supernatant. (**B**-**C**) Fluorescence microscope was used to take representative images of neuronal cells and measure axonal length. Actin was stained red and nuclei were stained blue with DAPI. Scale bar: 20 μm. (**D**) Scanning electron microscope was used to observe pyroptotic morphology of the cells. Red arrows represent bubble like cell protrusions. Scale bar: 10 μm. (**E**) The levels of LDH released from cells were measured by ELISA. (F-G) The Unc5b/PI3K/Akt/mTOR pathway-related proteins were detected by western blot. (H) Confocal microscope was used to observe the binding of netrin-1 to Unc5b. Netrin-1 was stained red, Unc5b was stained green, and yellow fluorescence was observed when the two were combined. The picture above shows the control group. The picture below shows that cells are only treated by EX-Netrin1. Scale bar: 2 μm. (EX-MSCs group: LPS+EX-MSCs group; EX-NC group: LPS+EX-NC group; EX-Netrin1 group: LPS+EX-Netrin1 group; LY294002 group: LPS+EX-Netrin1+LY294002 group; MHY1485 group: LPS+EX-Netrin1+LY294002+MHY1485 group. Error bars showed means ± SD; n = 3; *P < 0.05, **P < 0.01, ***P < 0.001, vs. EX-MSCs group; ^#^P < 0.05, ^##^P < 0.01, ^###^P < 0.001, vs. EX-NC group; ^P < 0.05, ^^P < 0.01, ^^^P < 0.001, vs. EX-Netrin1 group; ^$^P < 0.05, ^$$^P < 0.01, ^$$$^P < 0.001, vs. LY294002 group)

We further investigated whether the effect of EX-Netrin1 is realized through the PI3K/Akt/mTOR signalling pathway. In subsequent studies, the mTOR agonist MHY1485 and the PI3K inhibitor LY294002 were used. When PI3K was inhibited, the ability of EX-Netrin1 to attenuate the release of inflammatory factors was substantially attenuated, as measured by ELISA as elevated levels of inflammatory factors in the culture medium. However, when a mTOR agonist was used, inflammatory factors were significantly reduced (Fig. 4A). The PI3K inhibitors also impaired EX-Netrin1’s ability to promote axon growth, which was effectively restored when a mTOR agonist was used (Figs. 4B, C). When cells undergo pyroptosis, many small pores are formed on the cell membrane, and extracellular fluid will influx into the cell, causing the cell to swell into “bubbles”. Under scanning electron microscope, cells undergoing pyroptosis will form bleb like cell protrusions [46]. At the same time, lactate dehydrogenase (LDH) inside the cell also flows out to the outside through the small pores. By scanning electron microscopy, we found more bubbles in LPS-treated cells, indicating pyroptosis. EXs, especially EX-Netrin1, reduced the number of bubbles. However, when PI3K was inhibited, the number of bubbles increased again, and the mTOR agonist was able to abort this trend (Fig. 4D). Correspondingly, LDH release from the cells was increased in the LPS group and decreased in the EX-Netrin1 group. LDH release was increased by LY294002 but decreased by MHY1485 (Fig. 4E). In addition, we found that the signalling pathway was inhibited by LY294002, as indicated by decreased protein phosphorylation levels, and activated by MHY1485, but the signalling pathway had no remarkable impact on the expression of the receptor Unc5b (Figs. 4F-G). Finally, we used immunofluorescence to confirm that netrin-1 can bind to Unc5b. Confocal microscopy revealed that netrin-1 was stained red and that Unc5b was stained green, and yellow fluorescence was observed when the two were combined (Fig. 4H). In the control group, the fluorescence intensity of the two was weak and the combination was less. However, after the cells were treated with EX-Netrin1, the yellow fluorescence was significantly enhanced, indicating that the expression and binding of Unc5b and netrin-1 increased.

### The function of EX-netrin1 was attenuated by the inhibition of Unc5b but could be reversed by PI3K activation

**Fig. 5:**
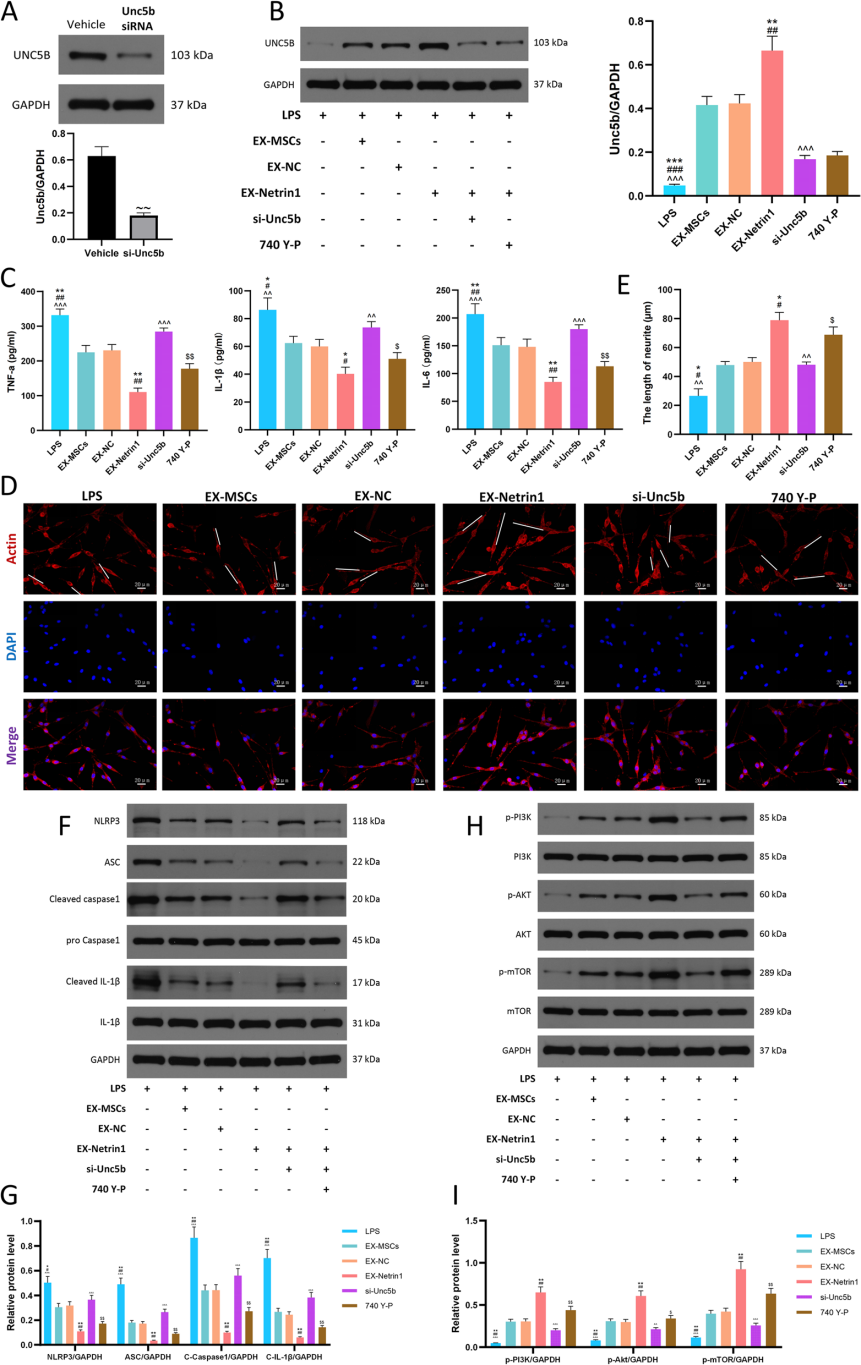
The function of EX-netrin1 was attenuated by the inhibition of Unc5b but could be reversed by PI3K activation. (**A**) Western blot showed that Unc5b was successfully inhibited by small interfering RNA (si-Unc5b). (**B**) Western blot suggested that transfection of si-Unc5b significantly mitigated the promoting effect of EX-Netrin1 on Unc5b expression in PC12 cells. (**C**) ELISA was performed to detect the contents of TNF-α , IL-1β and IL-6 in the cell supernatant. (**D**-**E**) Fluorescence microscope was used to take representative images of neuronal cells and measure axonal length. The white solid line represents the axon length. Scale bar: 20 μm. (**F**-**G**) Detection of pyroptosis-related proteins by western blot. (H-I) The PI3K/Akt/mTOR pathway-related proteins were detected by western blot. (EX-MSCs group: LPS+EX-MSCs group; EX-NC group: LPS+EX-NC group; EX-Netrin1 group: LPS+EX-Netrin1 group; si-Unc5b group: LPS+EX-Netrin1+si-Unc5b group; 740 Y-P group: LPS+EX-Netrin1+si-Unc5b+740 Y-P group. Error bars showed means ± SD; n = 3; ~~P < 0.01, vs. Vehicle group; *P < 0.05, **P < 0.01, ***P < 0.001, vs. EX-MSCs group; ^#^P < 0.05, ^##^P < 0.01, ^###^P < 0.001, vs. EX-NC group; ^P < 0.05, ^^P < 0.01, ^^^P < 0.001, vs. EX-Netrin1 group; ^$^P < 0.05, ^$$^P < 0.01, ^$$$^P < 0.001, vs. si-Unc5b group)

We further investigated the mechanism of EX-Netrin1 treatment in SCI by successfully inhibiting Unc5b expression with si-Unc5b (Fig. 5A). Meanwhile, si-Unc5b abolished the increase in the Unc5b receptor stimulated by EX-Netrin1, which could not be recovered even with the addition of the PI3K agonist 740 Y-P (Fig. 5B). In addition, when the number of receptors for netrin-1 was reduced, its anti-inflammatory effect was also greatly attenuated, and the PI3K agonist counteracted this phenomenon (Fig. 5C). Similar trends were observed for changes in axonal length (Figs. 5D, E). The same was true for pyroptosis, and the expression levels of pyroptosis-associated proteins were elevated by si-Unc5b and decreased by 740 Y-P administration (Figs. 5F, G). Not surprisingly, when Unc5b was knocked down, the PI3K/Akt/mTOR pathway was also inhibited, but 740 Y-P could restore it (Figs. 5H, I). This finding indicates that PI3K/Akt/mTOR is located downstream of Unc5b.

### EX-Netrin1 promoted functional recovery and nerve regeneration and attenuated inflammation and pyroptosis in SCI rats

**Fig. 6:**
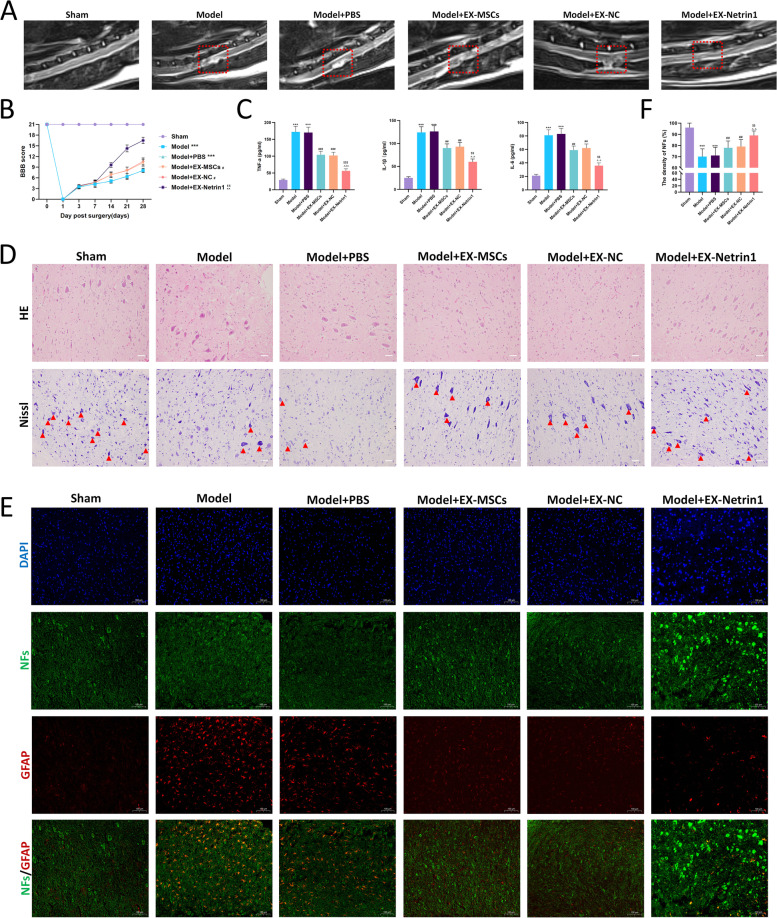
EX-Netrin1 improved functional recovery and reduced histopathological injury in SCI rats. (**A**) MRI was used to detect spinal cord injury in rats after 28 days. The red dotted box indicated the site of spinal cord injury. (**B**) BBB score was used to evaluate the functional recovery of hind limbs in rats. (**C**) After 28 days, spinal cord tissue was collected and ELISA was performed to detect the contents of TNF-α, IL-1β and IL-6 in spinal cord. (**D**) HE and Nissl staining of spinal cord tissues. Red arrows indicated Nissl bodies. Scale bar: 50 μm. (**E**-**F**) Representative images showing neurofilaments (NFs, green) and glial fibrillary acidic protein (GFAP, red) staining of spinal cord tissues, and (F) the density ratios of NFs. (Error bars showed means ± SD; n = 8. *P < 0.05, **P < 0.01, ***P < 0.001, vs. Sham group; ^#^P < 0.05, ^##^P < 0.01, ^###^P < 0.001, vs. Model+PBS group; ^P < 0.05, ^^P < 0.01, ^^^P < 0.001, vs. Model+EX-MSCs group; ^$^P < 0.05, ^$$^P < 0.01, ^$$$^P < 0.001, vs. Model+EX-NC group)

We explored the therapeutic effects of EX-Netrin1 in vivo by constructing a rat model of SCI. MRI was applied to examine spinal cord injury in rats after 28 days. The spinal cord of SCI rats was atrophic and thinned with hyperintense signal changes, which were recovered on imaging after EXs treatment (Fig. 6A, Fig. S4A). The BBB score was used to assess the recovery of hindlimb function in rats. Postoperative hindlimb function was more severely impaired in the Model group than in the Sham group, and recovery of hindlimb function was better in the EX group than in the Model+PBS group, with the EX-Netrin1 group showing the best recovery (Fig. 6B, Fig. S4B). The results suggested that injection of EX-Netrin1 might boost functional recovery in SCI rats. At 28 days after surgery, the levels of inflammatory factors in the model rats dramatically increased compared with those in the Sham group. After treatment with EXs, the levels of inflammatory factors declined markedly, and the effect of EX-Netrin1 was the best (Fig. 6C). According to the HE staining results, the spinal cord of the model rats had an incomplete structure and disordered tissue structure compared with the Sham group. In addition, inflammatory cell infiltration was evident. Injection of EXs could attenuate SCI, manifested by improved spinal cord tissue structure and a reduced number of inflammatory cells. Among them, the effect of EX-Netrin1 was the most obvious. The Nissl staining results were similar to those of HE staining. The results suggested that the number of Nissl bodies in the spinal cord tissues of the Model group was decreased compared with that in the Sham group. However, the number of Nissl bodies was increased by EXs treatment, and the neuroprotective effect of EX-Netrin1 was the most significant (Fig. 6D, Fig. S4C). Neurofilaments (NFs) are one of the indicators of neuronal regeneration, while glial fibrillary acidic protein-positive (GFAP+) astrocytes are one of the indicators for glial scars. Their relative density is widely used to evaluate the regeneration and recovery of nerve tissue [29, 47]. The density ratios of NFs to GFAP^+^ cells in the Model and Model+PBS groups were markedly decreased compared to those in the Sham group. When EXs treatment was used, the density of NFs rebounded. Especially in the EX-netrin1 group, the spinal cord tissue exhibited a high density of NFs (Figs. 6E, F).

**Fig. 7:**
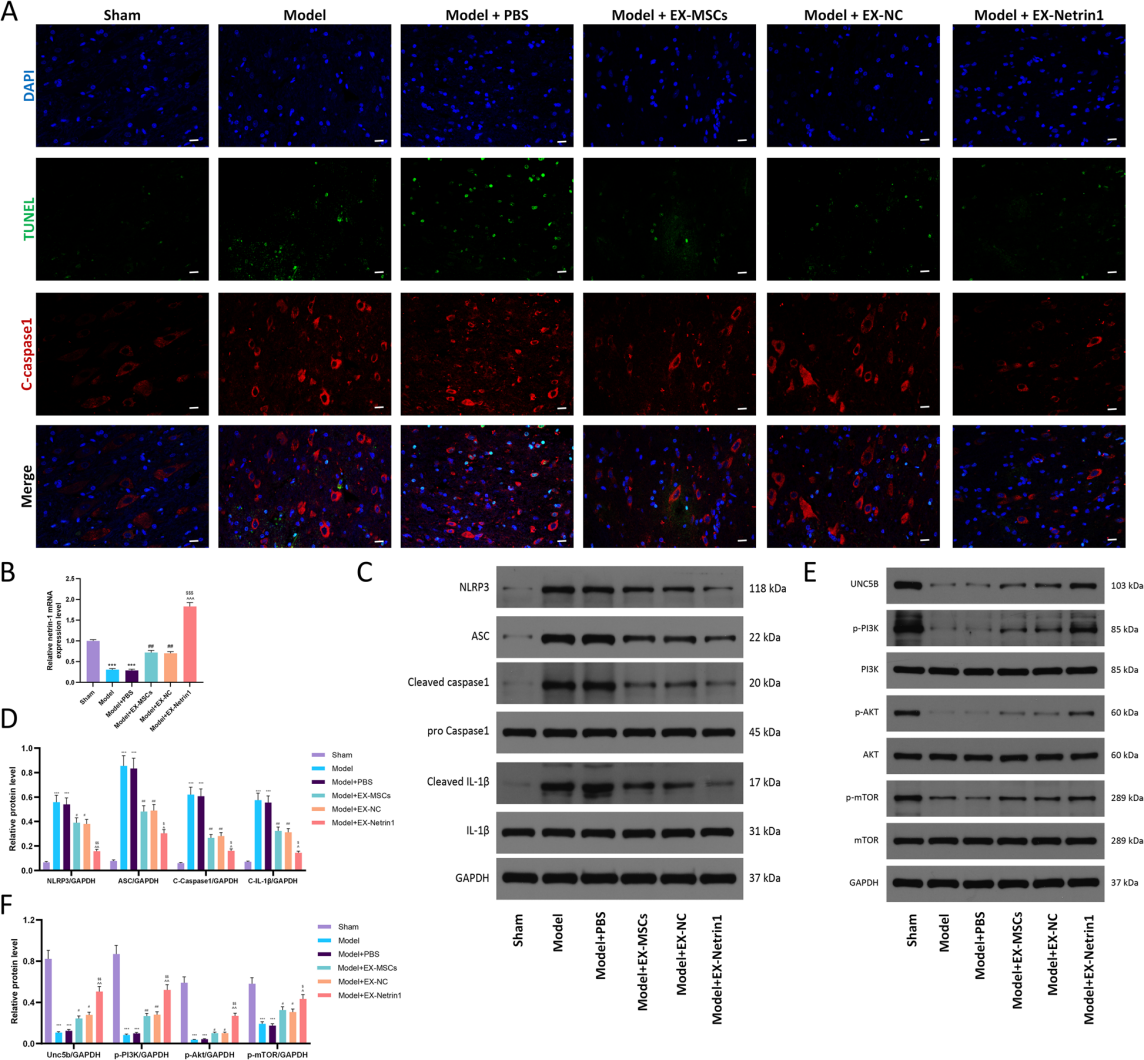
EX-Netrin1 improved functional recovery and reduced histopathological injury in SCI rats. (**A**) Representative fluorescence images showing TUNEL (green) and cleaved-caspase1 (red) double staining of spinal cord tissues. Scale bar: 20 μm. (B) The relative expression of netrin-1 mRNA in spinal cord tissues was detected by qRT-PCR. (**C**-**D**) Detection of pyroptosis-related proteins by western blot. (**E**-**F**) The PI3K/Akt/mTOR pathway-related proteins were detected by western blot. (Error bars showed means ± SD; n = 8. *P < 0.05, **P < 0.01, ***P < 0.001, vs. Sham group; ^#^P < 0.05, ^##^P < 0.01, ^###^P < 0.001, vs. Model+PBS group; ^P < 0.05, ^^P < 0.01, ^^^P < 0.001, vs. Model+EX-MSCs group; ^$^P < 0.05, ^$$^P < 0.01, ^$$$^P < 0.001, vs. Model+EX-NC group)

TUNEL (green) and cleaved-caspase1 immunofluorescence (red) double staining was used to evaluate neuronal pyroptosis. Fig. 7A shows that the Model group possessed a high level of pyroptosis and exhibited more green and red fluorescence, and this phenomenon could be inhibited by EXs; the effect of EX-Netrin1 was the strongest. In addition, we found that after SCI, netrin-1 mRNA in spinal cord tissue decreased, while EXs, especially EX-netrin1, elevated netrin-1 in spinal cord tissue of rats. This suggested that the engineered exosomes could effectively act on spinal cord tissue (Fig. 7B). Pyroptosis-associated proteins were measured by WB, and the results further indicated that EX-Netrin1 could inhibit pyroptosis after SCI in vivo (Figs. 7C, D). In terms of signalling pathways, the Unc5b/PI3K/AKT/mTOR axis in the Model group was notably inhibited. Injection of EXs could activate the axis, and the activation effect of EX-Netrin1 was the most obvious (Fig. 7E, F). This suggested that netrin-1 may exert its therapeutic effects on SCI in vivo through the Unc5b/PI3K/AKT/mTOR axis.

## Discussion

SCI is a devastating neurodegenerative disease that currently lacks clinically effective treatments, while nanotechnology and regenerative medicine strategies hold promise for the development of novel therapies. Stem cells can provide trophic support to neurons by replacing lost or damaged cells and improving the microenvironment of the spinal cord, thereby accelerating recovery of injured axons and accelerating SCI repair [48]. MSCs are found in multiple tissues, such as adipose, bone marrow, placenta and umbilical cord blood, and they are the most commonly used stem cells in animal studies and human clinical trials. Many studies have reported their multiple functions of homing, proliferation, differentiation, secretion and immune regulation [49]. EXs are nanoscale vesicles released outside the cell membrane that contain many small molecules, such as various nucleic acids, lipids, and proteins. The properties of these molecules are related to the cell of their origin [50].

The biological functions of EX-MSCs are similar to those of MSCs, but EX-MSCs are more stable and do not trigger an immune response in the body. Because of easy isolation, EX-MSCs could be used to deliver drugs or genetic materials to target tissues or cells. In addition, they are relatively small, so they can penetrate the blood-brain barrier and reach the damaged parts of the central nervous system [51, 52]. Besides, EX-MSCs contain various trophic and growth factors including brain-derived neurotrophic factor, insulin-like growth factor, hepatocyte growth factor, vascular endothelial growth facto r[53]. Therefore, EX-MSCs are a suitable choice for cell-free therapy. Multiple studies have revealed that EX-MSCs have good potential in SCI repair [11, 12, 44, 53]. These results were comparable to the results from this study in which we found that EXs had good effects in attenuating inflammation and pyroptosis and promoting axonal growth. Besides, EXs, especially EX-Netrin1, could promote the polarization of PC12 cells, which is manifested by the increase of axon growth and MAP2 expression. MAP2 mainly exists in the cell bodies and dendrites of neurons, and its content in dendrites is more than that in the cell bodies. As a marker protein of neurons, MAP2 plays an important role in the development, differentiation, shaping and polarity acquisition of neurons[54].

ModRNA is a novel gene vector that refers to mRNA synthesized in vitro using chemically modified bases. Compared with the conventional plasmid or viral gene vector, modRNA has the characteristics of high safety, [24] strong controllability, good stability, low immunogenicity and high protein translation ability [25–27]. It has shown potential in tissue regeneration,[55] gene therapy, [56] and vaccine research and development [57]. In this study, we explored a possible therapeutic approach for SCI by constructing modNetrin-1-enriched exosomes.

Netrin-1 was first discovered as an important guide factor for cell and axon migration during embryonic development. Later, it was found that it is also diffusely expressed in the mature nervous system [58]. Netrin-1’s effect is closely related to its receptors. The most common receptors of netrin-1 include those deleted in the colorectal cancer family (DCC) and the Unc5 homologous family. Among them, the family of Unc5 homologues includes four proteins, Unc5 a-d, and the most comprehensively studied is Unc5b, which plays an important role in inhibiting apoptosis, inhibiting inflammation and regulating angiogenesis [59, 60]. In this study, we found that LPS inhibited the expression of netrin-1. The underlying mechanism may be that in LPS-induced PC12 cells, inflammatory cytokines amplify the combination of NF-κB with the promoter of the netrin-1 gene [61], repressing netrin-1 transcription and the consequent netrin-1 protein synthesis. LPS was also found to inhibit netrin expression in vitro and in vivo by He et al. [62].

Attenuation of inflammation is one of the keys to SCI treatment. Unc5b, as a netrin-1 receptor that eliminates chemotaxis, is expressed on a variety of leukocyte membranes. Upon binding to unc5b, netrin-1 can inhibit the directional migration and accumulation of leukocytes from the vascular lumen to the site of inflammation [60]. Peroxisome proliferator-activated receptor gamma (PPARγ) is considered a downstream molecule of netrin-1 and is a key transcription factor regulating inflammation [63]. Netrin-1 activates PPARγ by binding to Unc5b and then activates PPARγ by inhibiting the nuclear transcription factor kappa B (NF-κB) pathway to reduce inflammatory factors [64].

This study found that PI3K/AKT/mTOR axis activation could inhibit the production of inflammatory factors. Inhibition of Unc5b expression aggravated the inflammatory response, but the use of a PI3K activator reversed this effect. In addition, in the presence of a PI3K inhibitor, activation of mTOR also reduced inflammation. This is in accordance with the study of Liu et al., who found that progesterone attenuated local inflammation in mice with intracerebral haemorrhage via activation of the PI3K/AKT/mTOR axis [22]. We also found that EX-Netrin1 reduced the release of inflammatory factors from microglia and promoted microglial M2 polarization. M2 polarized microglia can promote nerve regeneration after central nervous system injury [47]. Besides in neurological diseases, this pathway also involves the same anti-inflammatory effect in other systems, such as the skin and motor system [65, 66].

Axonal remodelling and regeneration are critical for neural network reconstruction after SCI. This process involves the expression and distribution of a series of related proteins, such as synaptophysin (SYP), postsynaptic density-95 (PSD-95), ras-related C3 botulinum toxin substrate (Rac1) and cell division cycle 42 (Cdc42). Zheng et al. [67] found that netrin-1 binding to DCC activated the JNK1/c-Jun pathway in the ischaemic penumbra of MCAO rats, increased SYP and PSD-95 expression levels in the periischaemic area, and promoted axon formation and regeneration. Shabani et al. [68] discovered that netrin-1 activates Cdc42 and Rac1 through DCC and promotes axon regeneration after nerve injury.

According to this study, when Unc5b was knocked down, the effect of netrin-1 on promoting axon growth was significantly weakened. Further research showed that 740 Y-P could counteract the influence of si-Unc5b. Simultaneously, when PI3K was inhibited, mTOR activation could still promote axon growth. The above results indicate that netrin-1 can activate the PI3K/AKT/mTOR pathway through the Unc5b receptor to accelerate axon growth. This is in accordance with the discovery of Zhu et al., who showed that catalpol activates the PI3K/AKT/mTOR axis and promotes the levels of downstream phosphorylates ribosomal protein S6 (p-S6) and growth associated protein 43 (GAP-43), thereby slowing axonal atrophy [21]. Neurons in a growing state need to synthesize extensive new proteins, and the expression level of intracellular p-S6 will be significantly upregulated. GAP-43 is mainly expressed in the growth cone terminals of neuronal axons and contributes to axon regeneration by inducing axonal sprouting and extension [69, 70].

Pyroptosis is a newly discovered form of programmed cell death accompanied by the generation of a variety of inflammatory factors, such as IL-1β and IL-18. Many studies have revealed that pyroptosis may play a more important role in SCI [71, 72]. The first step of the classical pyroptosis pathway is the formation of inflammatory bodies by pro-caspase-1, ASC and NLRP3. Then, pro-caspase-1 is cleaved to form caspase-1. Caspase-1 not only promotes the conversion of pro-IL-1β/18 to IL-1β/18 but also cleaves Gasdermin D (GSDMD) into two fragments. Its GSDMD -N-terminal part can form 10–15 nm small pores on the cell membrane, which eventually leads to the entry of water molecules and the leakage of cell contents [73].

The participation of the PI3K/AKT/mTOR axis in the regulation of pyroptosis has been found in a variety of diseases [74, 75]. However, the association of netrin-1 with pyroptosis has not been reported temporally. Based on our western blot analysis, LDH release assay and cell morphology observation, the pyroptosis rate rose markedly when Unc5b and PI3K were inhibited but decreased dramatically when PI3K and mTOR were activated. In this study, netrin-1 was found to reduce LPS-induced pyroptosis by activating the PI3K/AKT/mTOR pathway through the Unc5b receptor.

This study also has some limitations. First, we only studied the Unc5b receptor and ignored other receptors, such as DCC, which may also play a significant role in netrin-1 treatment for SCI. Second, in our study of netrin-1-induced axon growth, we only measured the length macroscopically but did not investigate p-S6, GAP-43 or other molecules microscopically. Finally, when performing animal experiments, we focused on investigating the therapeutic effects of EXs on SCI models without in-depth study of the PI3K/AKT/mTOR axis. Further research on these aspects is needed at a later date.

## Conclusions

**Fig. 8:**
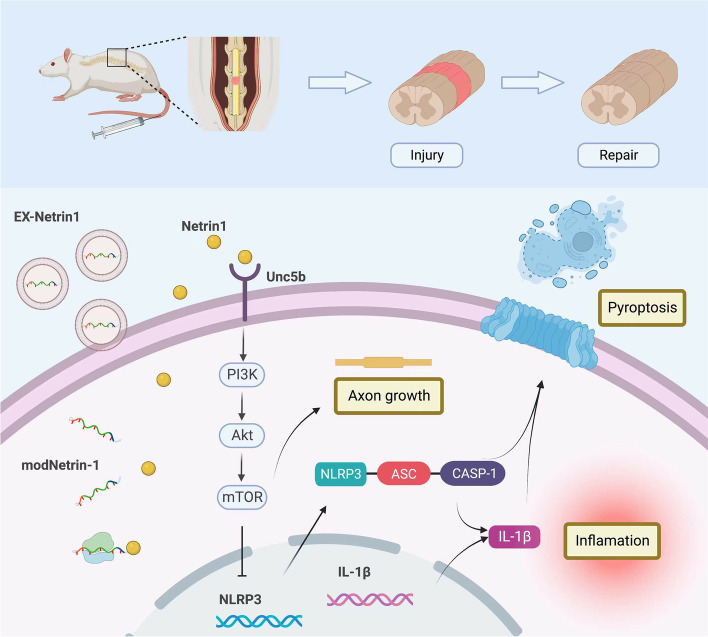
Graphical abstract. ModNetrin-1 enriched engineered exosomes attenuate inflammation and pyroptosis in spinal cord injury via the Unc5b/PI3K/Akt/mTOR pathway and promote axonal growth.

In this study, we transfected modRNA into MSCs to obtain engineered EX-netrin1. It was found that netrin-1 can mitigate the inflammatory response, reduce pyroptosis, and promote the growth of axons through the Unc5b/PI3K/AKT/mTOR pathway in vivo and in vitro (Fig. 8). In this study, we studied the molecular mechanism of EX-netrin1 in the treatment of SCI, providing a promising new method for the treatment of SCI.

## Supplementary Information

**Additional file 1.** Fig. S1. CCK8 assay for cell viability. (A) PC12 cell viability assay. (B) Microglia viability was examined under the same treatment conditions. (Error bars showed means ± SD; n = 3; *P < 0.05, **P < 0.01, ***P < 0.001, vs. Control group; ^#^P < 0.05, ^##^P < 0.01, ^###^P < 0.001, vs. LPS+EX-MSCs group; ^P < 0.05, ^^P < 0.01, ^^^P < 0.001, vs. LPS+EX-NC group; ^$^P < 0.05, ^$$^P < 0.01, ^$$$^P < 0.001, vs. LPS group). Fig. S2. EX-Netrin1 promoted the activation of PC12 cells. (A-B) WB was used to detect the expression of MAP2. (Error bars showed means ± SD; n = 3; ^*^P < 0.05, ^**^P < 0.01, ^***^P < 0.001, vs. Control group; ^#^P < 0.05, ^##^P < 0.01, ^###^P < 0.001, vs. LPS+EX-MSCs group; ^^^P < 0.05, ^^^^P < 0.01, ^^^^^P < 0.001, vs. LPS+EX-NC group; ^$^P < 0.05, ^$$^P < 0.01, ^$$$^P < 0.001, vs. LPS group). Fig. S3. EX-Netrin1 promoted HAPI rat microglia M2 polarization and inhibits pyroptosis. (A) ELISA was performed to detect the contents of TNF-α , IL-1β and IL-6 in the cell supernatant. (B) Immunofluorescence staining was used to detect M1 polarization marker protein CD86 and M2 polarization marker protein arginase-1 (Arg-1). Scale bar: 20 μm. (C) Fluorescence intensity of CD86 and Arg-1. (D) WB was used to detect the pyroptosis related protein NLRP3. (Error bars showed means ± SD; n = 3; ^*^P < 0.05, ^**^P < 0.01, ^***^P < 0.001, vs. Control group; ^#^P < 0.05, ^##^P < 0.01, ^###^P < 0.001, vs. LPS+EX-MSCs group; ^^^P < 0.05, ^^^^P < 0.01, ^^^^^P < 0.001, vs. LPS+EX-NC group; ^$^P < 0.05, ^$$^P < 0.01, ^$$$^P < 0.001, vs. LPS group). Fig. S4. Quantification of animal experiment data. (A) Quantification of the extent of spinal cord injury on MRI. Proportion of spinal cord high signal within 2mm of spinal cord injury site. (B) BBB score data of rats. (C) Number of nissl bodies per high magnification field of view. (Error bars showed means ± SD; n = 8. *P < 0.05, **P < 0.01, ***P < 0.001, vs. Sham group; ^#^P < 0.05, ^##^P < 0.01, ^###^P < 0.001, vs. Model+PBS group; ^P < 0.05, ^^P < 0.01, ^^^P < 0.001, vs. Model+EX-MSCs group; ^$^P < 0.05, ^$$^P < 0.01, ^$$$^P < 0.001, vs. Model+EX-NC group)

## Data Availability

The datasets used and/or analysed during the current study are available from the corresponding author on reasonable request.

## Abbreviations

SCI: spinal cord injury
EX: exosomes
PI3K: phosphatidylinositol-3-kinase
AKT: protein kinase B
mTOR: mammalian target of rapamycin
MSCs: mesenchymal stem cells
modRNA: modified message RNA
GFP: green fluorescent protein
qRT–PCR: quantitative real-time polymerase chain reaction
NLRP3: nucleotide-binding oligomerization domain-like receptor protein 3
ASC: apoptosis-associated speck-like protein
NTA: nanoparticle tracking analysis
TEM: transmission electron microscopy
DAPI: 4–6-diamidino-2-phenylindole
siRNA: small interfering RNA
LPS: lipopolysaccharide
ELISA: enzyme-linked immunosorbent assay
NF: neurofilament
GFAP: glial fibrillary acidic protein
LDH: lactate dehydrogenase
BBB: Basso, Beattie & Bresnahan
HE: haematoxylin and eosin
DCC: deleted in colorectal cancer family
PPARγ: peroxisome proliferator-activated receptor gamma
NF-κB: nuclear transcription factors kappa B
SYP: synaptophysin
PSD-95: postsynaptic density-95
Rac1: ras-related C3 botulinum toxin substrate
Cdc42: cell division cycle 42
p-S6: phosphorylates ribosomal protein S6
GAP-43: growth associated protein 43
GSDMD: gasdermin D
NGF: nerve growth factor
MAP2: microtubule-associated protein 2.
