# Supplementary material for: Engineered exosomes enriched in netrin-1 modRNA promote axonal growth in spinal cord injury by attenuating inflammation and pyroptosis
Source: Biomater Res. 2023 Jan 17;27:3. doi: 10.1186/s40824-023-00339-0 (PMC9843879; doi:10.1186/s40824-023-00339-0)
Supplement: Supplementary file 1 — Additional file 1. Fig. S1. CCK8 assay for cell viability. (A) PC12 cell viability assay. (B) Microglia viability was examined under the same treatment conditions. (Error bars showed means ± SD; n = 3; *P < 0.05, **P < 0.01, ***P < 0.001, vs. Control group; #P < 0.05, ##P < 0.01, ###P < 0.001, vs. LPS+EX-MSCs group; ^P < 0.05, ^^P < 0.01, ^^^P < 0.001, vs. LPS+EX-NC group; $P < 0.05, $$P < 0.01, $$$P < 0.001, vs. LPS group). Fig. S2. EX-Netrin1 promoted the activation of PC12 cells. (A-B) WB was used to detect the expression of MAP2. (Error bars showed means ± SD; n = 3; *P < 0.05, **P < 0.01, ***P < 0.001, vs. Control group; #P < 0.05, ##P < 0.01, ###P < 0.001, vs. LPS+EX-MSCs group; ^P < 0.05, ^^P < 0.01, ^^^P < 0.001, vs. LPS+EX-NC group; $P < 0.05, $$P < 0.01, $$$P < 0.001, vs. LPS group). Fig. S3. EX-Netrin1 promoted HAPI rat microglia M2 polarization and inhibits pyroptosis. (A) ELISA was performed to detect the contents of TNF-α , IL-1β and IL-6 in the cell supernatant. (B) Immunofluorescence staining was used to detect M1 polarization marker protein CD86 and M2 polarization marker protein arginase-1 (Arg-1). Scale bar: 20 μm. (C) Fluorescence intensity of CD86 and Arg-1. (D) WB was used to detect the pyroptosis related protein NLRP3. (Error bars showed means ± SD; n = 3; *P < 0.05, **P < 0.01, ***P < 0.001, vs. Control group; #P < 0.05, ##P < 0.01, ###P < 0.001, vs. LPS+EX-MSCs group; ^P < 0.05, ^^P < 0.01, ^^^P < 0.001, vs. LPS+EX-NC group; $P < 0.05, $$P < 0.01, $$$P < 0.001, vs. LPS group). Fig. S4. Quantification of animal experiment data. (A) Quantification of the extent of spinal cord injury on MRI. Proportion of spinal cord high signal within 2mm of spinal cord injury site. (B) BBB score data of rats. (C) Number of nissl bodies per high magnification field of view. (Error bars showed means ± SD; n = 8. *P < 0.05, **P < 0.01, ***P < 0.001, vs. Sham group; #P < 0.05, ##P < 0.01, ###P < 0.001, vs. Model+PBS group; ^P < 0.05, ^^P < 0.01, ^^^P < 0.001, vs. [file 40824_2023_339_MOESM1_ESM.pdf]

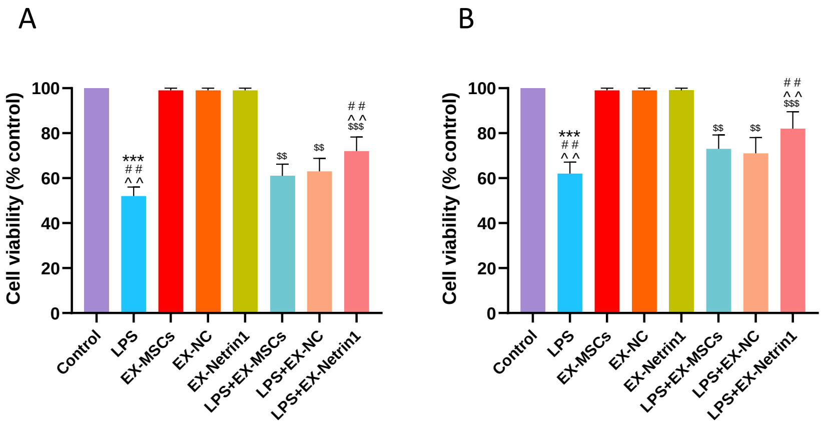

Fig. S1. CCK8 assay for cell viability. (A) PC12 cell viability assay. (B) Microglia viability was examined under the same treatment conditions. (Error bars showed means ± SD; n = 3; *P < 0.05, **P < 0.01, ***P < 0.001, vs. Control group; $^{\#}P < 0.05$, $^{\#\#}P < 0.01$, $^{\#\#\#}P < 0.001$, vs. LPS+EX-MSCs group; ^P < 0.05, ^^P < 0.01, ^^^P < 0.001, vs. LPS+EX-NC group; $^{\$}P < 0.05$, $^{\$\$}P < 0.01$, $^{\$\$\$}P < 0.001$, vs. LPS group)

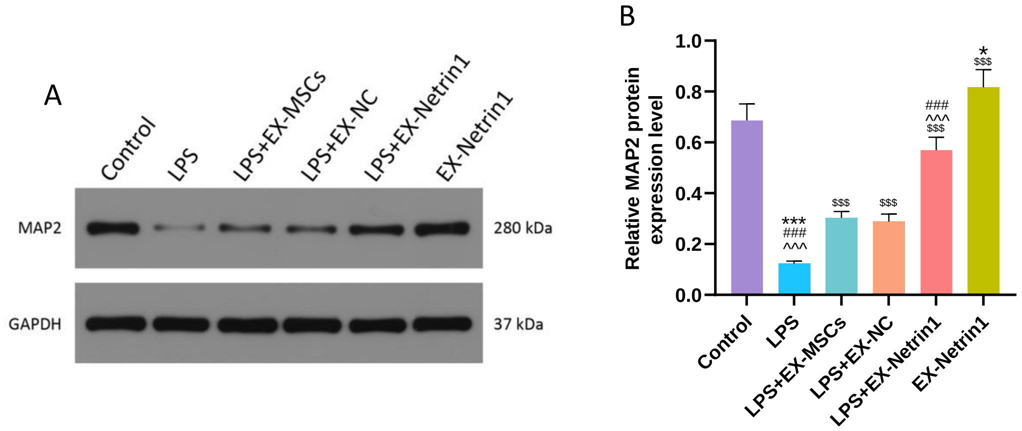

Fig. S2. EX-Netrin1 promoted the activation of PC12 cells. (A-B) WB was used to detect the expression of MAP2. (Error bars showed means ± SD; n = 3; *P < 0.05, **P < 0.01, ***P < 0.001, vs. Control group; $^{\#}$P < 0.05, $^{\#\#}$P < 0.01, $^{\#\#\#}$P < 0.001, vs. LPS+EX-MSCs group; $^{\wedge}$P < 0.05, $^{\wedge\wedge}$P < 0.01, $^{\wedge\wedge\wedge}$P < 0.001, vs. LPS+EX-NC group; $^{\$}$P < 0.05, $^{\$\$}$P < 0.01, $^{\$\$\$}$P < 0.001, vs. LPS group)

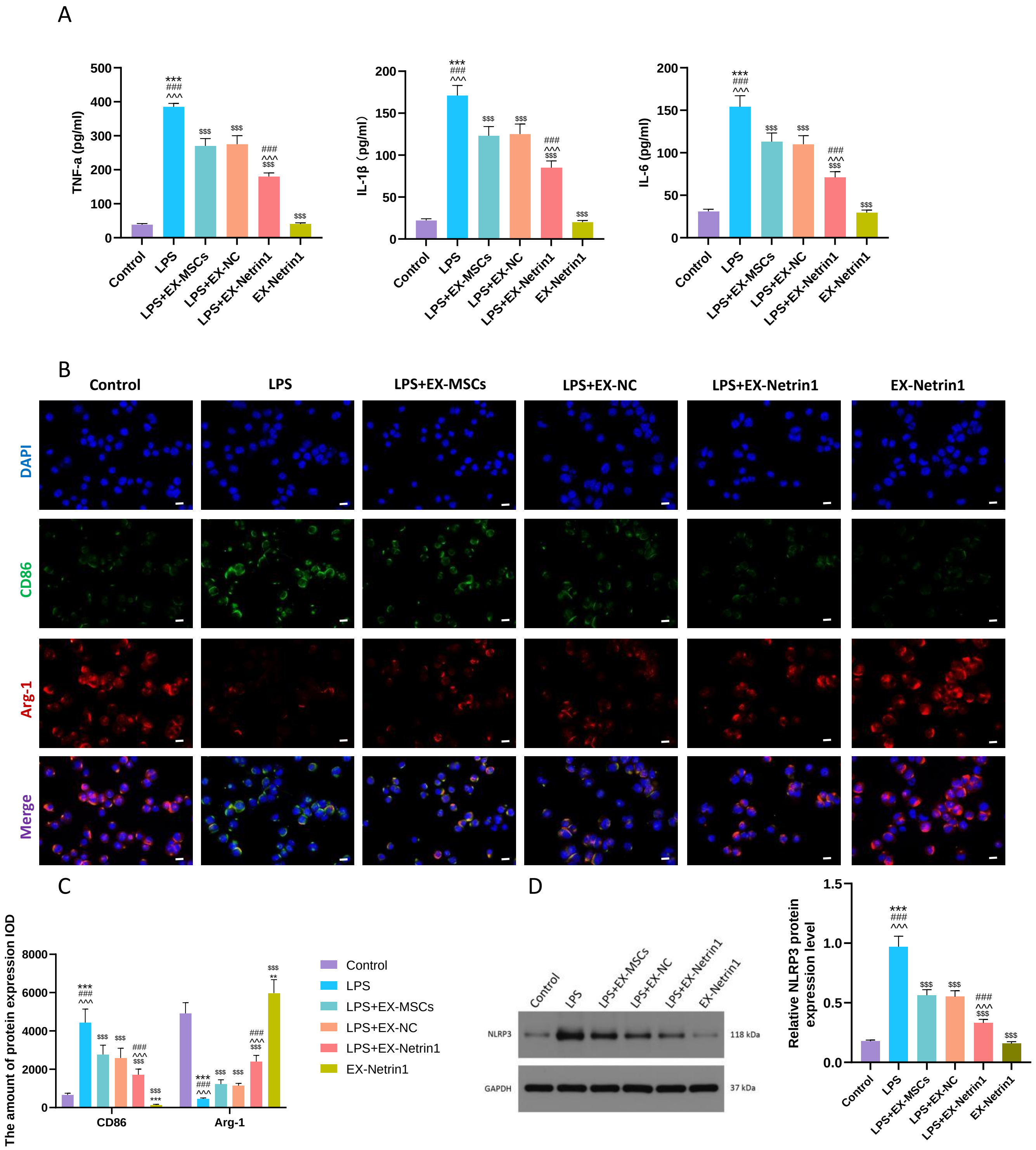

Fig. S3. EX-Netrin1 promoted HAPI rat microglia M2 polarization and inhibits pyroptosis. (A) ELISA was performed to detect the contents of TNF-α , IL-1β and IL-6 in the cell supernatant. (B) Immunofluorescence staining was used to detect M1 polarization marker protein CD86 and M2 polarization marker protein arginase-1 (Arg-1). Scale bar: 20 μm. (C) Fluorescence intensity of CD86 and Arg-1. (D) WB was used to detect the pyroptosis related protein NLRP3. (Error bars showed means ± SD; n = 3; $^{*}P < 0.05$, $^{**}P < 0.01$, $^{***}P < 0.001$, vs. Control group; $^{\#}P < 0.05$, $^{\#\#}P < 0.01$, $^{\#\#\#}P < 0.001$, vs. LPS+EX-MSCs group; $^{\wedge}P < 0.05$, $^{\wedge\wedge}P < 0.01$, $^{\wedge\wedge\wedge}P < 0.001$, vs. LPS+EX-NC group; $^{\$}P < 0.05$, $^{\$\$}P < 0.01$, $^{\$\$\$}P < 0.001$, vs. LPS group)

A

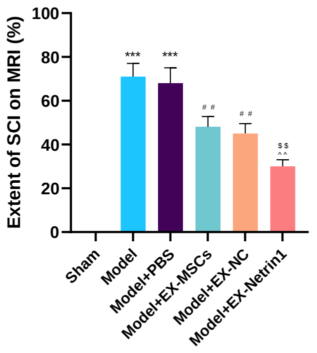

C

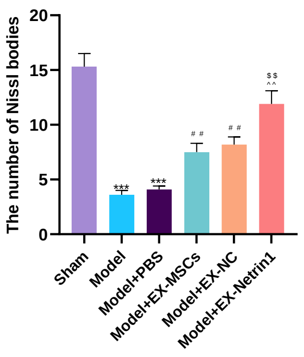

B

Day	Sham	Model	Model+PBS	Model+EX-MSCs	Model+EX-NC	Model+EX-Netrin1
0	21	21	21	21	21	21
1	21	0	0	0	0	0
3	21	3.50±0.50	3.37±0.48	3.62±0.48	3.62±0.48	3.75±0.43
7	21	4.25±0.96	4.37±0.85	4.62±0.69	4.75±0.66	4.75±0.44
14	21	5.12±0.33	5.00±0.50	6.87±0.59	6.87±0.78	9.62±0.69
21	21	6.37±0.85	6.50±0.70	8.00±0.10	8.12±0.92	14.25±0.96
28	21	8.12±0.59	8.00±0.50	10.25±0.96	10.62±0.99	16.50±0.88

Fig. S4. Quantification of animal experiment data. (A) Quantification of the extent of spinal cord injury on MRI. Proportion of spinal cord high signal within 2mm of spinal cord injury site. (B) BBB score data of rats. (C) Number of nissl bodies per high magnification field of view. (Error bars showed means ± SD; n = 8. *P < 0.05, **P < 0.01, ***P < 0.001, vs. Sham group; $^{\#}P < 0.05$, $^{\#\#}P < 0.01$, $^{\#\#\#}P < 0.001$, vs. Model+PBS group; ^P < 0.05, ^^P < 0.01, ^^^P < 0.001, vs. Model+EX-MSCs group; $^{\$}P < 0.05$, $^{\$\$}P < 0.01$, $^{\$\$\$}P < 0.001$, vs. Model+EX-NC group)
